# Dimensions of psychological flexibility and their significance in people with somatic symptoms: The 18-item Flexibility Index Test (FIT-18)

**DOI:** 10.1177/13591053241239129

**Published:** 2024-04-02

**Authors:** Tim Y Koppert, Renée van Hoek, Rinie Geenen

**Affiliations:** 1 Leiden University, The Netherlands; 2 Altrecht Psychosomatic Medicine, The Netherlands; 3 Utrecht University, The Netherlands

**Keywords:** fatigue, mental well-being, pain, physical functioning, psychological flexibility

## Abstract

Psychological flexibility has been hypothesized to preserve health in bad times. We examined whether psychological flexibility as assessed with an abbreviated questionnaire, was indicated to preserve mental and physical health when having somatic symptoms. Principal axis factoring indicated that two dimensions best represented the 60-item Flexibility Index Test (FIT-60) questionnaire: “mindfulness and acceptance” (M&A) and “commitment and behavior change” (C&BC). We selected 18 items that best denoted these dimensions (FIT-18 questionnaire). Regression analyses in 2060 Dutch people with and without persistent somatic symptoms, indicated that the M&A dimension (β = 0.33, *p* < 0.001) and C&BC dimension (β = 0.09, *p* < 0.001) were additively associated with mental well-being, but not with physical functioning. Moreover, the M&A dimension was indicated to protect mental well-being when having more severe somatic symptoms (β = 0.11, *p* < 0.001). The observed differential associations with health suggest the significance for health of the two dimensions of psychological flexibility as assessed with the FIT-18 questionnaire.

## Introduction

Psychological flexibility refers to approaching difficult or challenging internal states (thoughts, emotions, and bodily sensations) in a non-judgmental, mindful way, and being committed to pursue one’s values (Hayes et al., 2006; Kashdan and Rottenberg, 2010). It is considered key to preserve well-being and functioning when confronted with adversities (Hayes et al., 2012; Presti et al., 2020). Also, for people with somatic symptoms, psychological flexibility may help to preserve well-being and functioning. Acceptance and commitment therapy (ACT) is the treatment of choice for enhancing psychological flexibility and has been indicated to enhance well-being and functioning in people with persistent somatic symptoms (Hughes et al., 2017; Maunick et al., 2023). A brief assessment of psychological flexibility is important to be able to examine who will benefit from ACT and to monitor psychological flexibility as a mediator or outcome. This study examined the potential significance of an abbreviated version of an existing questionnaire of psychological flexibility in people with persistent somatic symptoms.

The theoretical (hexaflex) model of psychological flexibility postulates that the construct includes six mutually interconnected processes, that depend on each other and are difficult to entangle (Hayes et al., 2006): “acceptance” (willingness to experience uncomfortable thoughts and feelings without attempts to change or avoid them), “cognitive defusion” (distancing oneself from unhelpful thoughts and noticing they are not facts that need to be acted upon), “contact with the present moment” (ongoing non-judgmental contact with thoughts, feelings and other private events as they occur), “self-as-context” (taking an observer perspective toward one’s own experiences), “values” (chosen life directions that guide behavior), and “committed action” (engaging in value-based behavior). Based on theoretical and clinical considerations, these six processes have been further organized into two overarching processes (Hayes et al., 2006): “acceptance” and “cognitive defusion” into a “mindfulness and acceptance” process; “values” and “committed action” into a “commitment and behavior change” process. “Contact with the present moment” and “self-as-context” are represented in both processes, but when a dichotomy is created in research, they are usually grouped under the “mindfulness and acceptance” process (Morin et al., 2021).

In clinical practice and the workplace, each of the six processes, as supplemented by traditional functional analysis, and applied to the specific cognitive, behavioral, emotional, and social content, can be linked to intervention methods and clinical targets (Bond et al., 2015; Hayes et al., 2011, 2012). This is one of the reasons why researchers have tried to assess psychological flexibility and its processes by self-report questionnaires. Dimensions that summarize items from self-report questionnaires show the more enduring traits or skills that vary between subjects. These factors or dimensions may reflect but are not similar to the assumed underlying processes. Self-reported assessments can be used to screen whether a person may need a more mindfulness and acceptance-based or value-based therapeutic approach, to examine moderation and mediation by these processes, or to monitor and evaluate the outcome of therapy. Self-report questionnaires have been developed to measure a single process and two to five processes of psychological flexibility, or a single overarching dimension (Baer et al., 2006; Bond et al., 2011; Gámez et al., 2011; Gillanders et al., 2014). In the Netherlands, the Flexibility Index Test (FIT-60) was developed to measure all six processes of psychological flexibility using 10 items for each process (Batink et al., 2012).

The initial study into the psychometric qualities of the FIT-60 indicated acceptable to good internal consistencies and sensitivity to change of the six dimensions (Batink et al., 2012). Later studies observed associations of psychological flexibility with personality traits, mental well-being, and somatic symptoms (Koppert et al., 2021a, 2021b; Pakenham et al., 2024; Steenhaut et al., 2020). However, these later studies only examined the overarching dimension of psychological flexibility, not the six separate processes. Because factor analyses were not applied, it remained unclear whether the FIT-60 consistently reflected the six processes. Quantitative assessment of all six processes in clinical practice and research using dimensions on self-report questionnaires requires high inter-item correlations within a process and low correlations between processes. This can be determined in factor analysis. If factor analysis shows that less than six dimensions are reflected by the FIT-60 questionnaire, than the number of items of this questionnaire can be reduced. There are also practical considerations to reduce the number of items of the FIT-60: respondents commented that the questionnaire is too long and that several items are difficult to understand.

Having a brief measure of psychological flexibility is useful from a practical point of view, but its development should not be at the cost of its contents. Psychological flexibility is considered particularly useful when challenges arise that produce distress and hamper goal pursuit (Doorley et al., 2020). This implies that more psychological flexibility is expected to be associated with better mental well-being and physical functioning (Hayes et al., 2006), and more specifically that the interaction of somatic symptom severity with psychological flexibility is significantly associated with these health variables. If the significant interaction shows that in case of higher psychological flexibility the association of somatic symptoms with health variables is lower, then a health-protecting role of psychological flexibility is indicated.

The aim of our study was to examine whether psychological flexibility as assessed with an abbreviated FIT-60 questionnaire, was indicated to preserve mental and physical health when having somatic symptoms. To that aim, we first examined how many dimensions can be distinguished with the FIT-60, and second, whether the resulting dimensions of psychological flexibility were associated with mental well-being and physical functioning, and whether the association of somatic symptom severity with mental well-being and physical functioning was weaker in case of higher psychological flexibility.

## Methods

### Participants

This study involves a new analysis of data that have been described previously (Koppert et al., 2021a, 2021b). Data from two separate online surveys in the general, Dutch-speaking adult (≥18 years) population were analyzed. The first data collection was from November 2018 to May 2019 and the second from March to May 2020. The aim of the first data collection as explained to the participants was to get insight into the association of well-being and somatic symptoms with psychological flexibility. The aim of the second data collection as presented to participants was to get insight into the psychological response to the COVID-19 crisis. In both samples, we retained all participants with complete assessments on psychological flexibility as well as on the questionnaire that assessed somatic symptom severity, mental well-being, and physical functioning. Figure S1 (Supplemental Material) shows the flowchart comprising both samples. A total of 2739 participants started to fill out the online questionnaire. Of this group, 679 (24.8%) participants did not complete the entire survey. The dropouts differed from the completers (*n* = 2060), in terms of age (mean age [SD] for dropouts 45.0 [15.8] and for completers 47.7 [14.8], gender (*n* = 121 [17.8%] men vs 558 [82.2%] women and *n* = 412 [20.0%] men vs 1648 [80.0%] women, χ^2^ = 7.54, *p* = 0.02) and education level (*n* = 311 [46.5%] lower vs *n* = 358 [53.5%] higher and *n* = 762 [37.2%] lower vs *n* = 1287 [62.8%] higher, χ^2^ = 18.25, *p* < 0.001).

### Procedure

Participants were recruited through social media (e.g. Facebook, Instagram, LinkedIn, local internet sites) and websites of patient associations with a focus on associations for people with persistent somatic symptoms. This recruitment note was shared by other individuals and groups. Participants filled out the online survey at a secure university website. A hyperlink to the online survey was provided, where participants were informed about the study and could provide informed consent, after which they were allowed to participate. They were not compensated for their participation. Approval for the two data collections was given by the Ethics Committee of the Faculty of Social and Behavioral Sciences of Utrecht University, The Netherlands (FETC17-120 and FETC20-190).

### Instruments

#### Psychological flexibility

The FIT-60 was used to measure psychological flexibility (Batink et al., 2012). This instrument includes 10 items for each of the six processes of psychological flexibility (Hayes et al., 2006). Items are based on a literature review of psychological flexibility and on four existing questionnaires. The Acceptance and Action Questionnaire (AAQ-II, Bond et al., 2011) was used to measure “acceptance,” the Cognitive Fusion Questionnaire (CFQ-13, Gillanders et al., 2014) to assess “cognitive defusion,” the Five Facet Mindfulness Questionnaire (FFMQ, Baer et al., 2006) to assess “contact with the present moment,” and the Value Living Questionnaire (VLQ-2, Wilson et al., 2010) to assess “values.” Participants indicated to what extent an item applies to them on a 7-point Likert scale, ranging from 0 (“totally disagree”) to 6 (“totally agree”). The range of the total score is from 0 to 360 (Batink et al., 2012). Higher scores denote more flexibility.

The initial psychometric qualities of the FIT-60 showed acceptable to good internal consistencies for all scales but self-as-context (that had a questionable value). Cronbach’s alphas were 0.84 (acceptance), 0.87 (cognitive defusion), 0.78 (contact with the present moment), 0.69 (self-as-context), 0.78 (values), and 0.84 (committed action); Cronbach’s alpha for the total dimension was 0.95 (Batink et al., 2012). In our current study, Cronbach’s alpha was poor for self-as-context and acceptable to good for the other dimensions reflecting the six processes during the two occasions: 0.84 and 0.83 (acceptance), 0.88 and 0.90 (cognitive defusion), 0.81 and 0.84 (contact with the present moment), 0.56 and 0.53 (self-as-context), 0.78 and 0.79 (values), and 0.83 and 0.86 (committed action). Cronbach’s alpha of the 60-item total dimension was 0.95.

#### Somatic symptom severity

The severity of somatic symptoms was measured with the pain and vitality scales of the Dutch version of the 36-item RAND Short Form Health Survey (RAND SF-36, Vander Zee et al., 1996). The pain scale consists of 4 items assessing the level of bodily pain and its interference with daily activities during the past 4 weeks, on 6- and 5-point Likert scales. The vitality scale consists of 4 items assessing the level of fatigue and energy during the past 4 weeks on 6-point Likert scales. After reversing the scores, higher scores on the SF-36 reflect more severe pain and fatigue. We used the standardized mean deviation from the norm scores (Vander Zee et al., 1996) of these pain and fatigue scales as a measure of somatic symptom severity. In this study, Cronbach’s alpha of this 2-scale composite score was 0.76.

#### Mental well-being and physical functioning

Also, mental well-being and physical functioning were measured with the RAND SF-36. As an indicator of mental well-being, we used the mean of the standardized mean deviation from the norm scores (Vander Zee et al., 1996) of three subscales: emotional well-being, role limitations due to emotional problems, and social functioning. Physical functioning was derived in the same way using three subscales: physical functioning, role limitations due to physical problems, and general health. Higher mental well-being and physical functioning scores reflect better health. In the current study, Cronbach’s alpha of these 3-scale composite scores were 0.76 for mental well-being and 0.82 for physical functioning.

### Statistical analyses

Statistical analyses were performed using IBM SPSS statistics version 29. Tests were two-tailed and statistical significance was considered for *p* < 0.05. To get insight into the number of dimensions of psychological flexibility as measured with the FIT-60, we employed exploratory factor analyses for one-half of the data. To verify the fit of the resulting factor structure, confirmatory factor analysis was used for the other half of the data. In exploratory analysis, principal axis factoring with direct oblimin rotation was used because the items were expected to correlate (Hayes et al., 2006). The number of factors was determined based on the scree-plot, the pattern of factor loadings, internal consistency of the items within a factor, the number of items included in a factor (a minimum of 3), and the content of the items. Criteria for excluding an item from further analysis were a factor loading below 0.45 on any factor (Comrey and Lee, 1992) or a factor loading above 0.32 on two or more factors (Costello and Osborne, 2005). Cronbach’s alpha was used to analyze the internal consistency.

In constructing a shorter version of the FIT-60, to preserve diversity of the items, we wanted to include at least three items of each process in a dimension reflecting that process. In selecting items, we first took account of the factor loadings (high on the primary factor and low on other factors). Items that had a too large conceptual overlap with other items were not chosen. Also, items that were difficult to understand for respondents were not chosen.

To confirm the two-factor structure of the FIT-18, confirmatory factor analysis was applied using IBM SPSS AMOS 29. Maximum likelihood estimation was chosen to estimate the structural equation model. To examine goodness of fit, we followed Hooper et al. (2008) by reporting the Chi-Square statistic, its degrees of freedom and *p*-value, the root mean square error of approximation (RMSEA) and its associated confidence interval, the standardized root mean square residual (SRMR), the Comparative Fix Index (CFI), and the Parsimony-Adjusted Measures Index (PNFI). Acceptable model fits were defined as: a sample-size adjusted χ^2^-value (χ^2^/df)<1 (upper limit 5), an RMSEA close to 0.06 (upper limit 0.07), an SRMR < 0.05 (upper limit 0.08), a CFI ≥ 0.95 (lower limit ≥ 0.90), and a PNFI value > 0.50.

Pearson’s correlations were calculated to assess the univariate associations between the resulting psychological flexibility dimensions of the shortened FIT-60 with demographic variables, symptom severity, mental well-being, and physical functioning.

Moderator analyses were done to examine whether and which dimensions of psychological flexibility were indicated to preserve mental well-being and physical functioning in case of somatic symptoms. Continuous predictor variables were centered and interactions were computed from these centered variables. In linear regression analysis, mental well-being and physical functioning were associated with symptom severity, the psychological flexibility dimensions, and the interaction between symptom severity and the psychological flexibility dimensions; age, gender, and education level were added to the model as covariates. To interpret significant interactions, regression lines for individuals with low (−1 SD) and high (+1 SD) scores on the interacting variables were plotted (Aiken and West, 1991). The magnitude of the interaction was indicated with Cohen’s *d* effect sizes, with values of 0.20, 0.50, and 0.80 as cutoffs for small, medium, and large effects, respectively (Cohen, 1992).

## Results

### Participant characteristics

Table 1 shows the characteristics of participants. The sample that included 2060 participants showed a predominance of women and a large representation of people with persistent somatic symptoms, such as people with an inflammatory rheumatic disease (11.6%), central sensitivity syndrome (30.2%), and osteoarthritis (11.7%). These chronic illnesses often have a concurrent other disease.

**Table 1.** Characteristics of participants (*N* = 2060).

Variable	Statistic
Age (years)	
Mean (SD)	47.7 (14.8)
Range	18–91
Gender, *n* (%)	
Men	412 (20.0)
Women	1648 (80.0)
Education level, *n* (%)^a^	
Low	762 (37.0)
High	1287 (62.5)
Missing	11 (0.5)
Marital status, *n* (%)	
Single	618 (30.0)
In a relation	1386 (67.3)
Unknown	56 (2.7)
Type of illness, *n* (%)	
Not any illness	583 (28.3)
Inflammatory rheumatic disease^b^	239 (11.6)
Central sensitivity syndrome^c^	622 (30.2)
Osteoarthritis	241 (11.7)
Pulmonary	274 (13.3)
Skin	100 (4.9)
Cancer	46 (2.2)
Cardiovascular	286 (13.9)
Psychiatric	266 (12.9)
Neurological	166 (8.1)
Obesity	198 (9.6)
One or more other non-listed diseases	205 (9.9)
Health (RAND SF-36), Mean (SD)^d^	
Mental well-being (RAND SF-36), Mean (SD)^d^	−0.58 (1.01)
Physical functioning (RAND SF-36), Mean (SD)^d^	−0.46 (0.96)
Somatic symptom severity (RAND SF-36), Mean (SD)^d^	0.50 (0.97)
Psychological flexibility (FIT-60), Mean (SD)^e^	228.6 (49.1)

a Education level: low: lower general secondary education or lower; high: higher general secondary education or higher.

b These participants reported to have a chronic rheumatic disease other than osteoarthritis or fibromyalgia.

c This group comprises participants with fibromyalgia, chronic fatigue syndrome (CFS), irritable bowel syndrome (IBS), somatoform disorder/somatic symptom disorder, chronic headache (not migraine), or chronic pain elsewhere in the body (not the head).

d This score is the mean of standardized deviation scores from the general adult population norm (Vander Zee et al., 1996). Scores for somatic symptom severity were reversed: higher scores reflect more pain and fatigue.

e This total score ranges from 0 to 360, with higher scores reflecting more psychological flexibility.

### Reduction of the size of the questionnaire

#### Six-factor solution

Although the scree-plot of factor analysis clearly indicated a two-factor solution, we explored whether factor analysis would show the six processes of psychological flexibility when forcing a six-factor solution. The total variance explained by the six factors after principal axis factoring with oblique rotation was 42.0%. Table S1 in the Supplemental File shows the pattern matrix after principal axis factoring. All six factors included at least three items with a high factor loading >0.45. Factor 1 included 11 items representing three processes (all items had a negative wording): “Contact with the present moment,” “Acceptance,” and “Cognitive defusion.” Factor 2 included seven “Committed action” items and one “Values” item. Factor 3 included three “Contact with the present moment” items, all with a positive wording. Factor 4 was a uniform “Values” factor including five items. Factor 5 (six items) and factor 6 (three items) both included items representing two processes. As the six processes were neither clearly represented in the six factors solution, nor in three to five-factor solutions, we tried a two-factor solution.

#### Two-factor solution

Principal axis factoring yielded a clearly interpretable two-factor solution (see Table S2 in the Supplemental File). The eigenvalues of the two factors were 16.8 (28.0%) and 3.3 (5.5%) with a total explained variance of 33.5%. The first factor included 25 items with high (>0.45) loadings and low (<0.32) cross-loadings; item 3 showed a significant but negative loading on factor 1 and was therefore not included in this factor. The resulting 24 items comprised four processes. These are “acceptance” and “cognitive defusion,” that were described in the theoretical model (Hayes et al., 2006) under the “mindfulness and acceptance” label as well as “contact with the present moment” and “self-as-context” that have been grouped under this label in empirical research (Morin et al., 2021). We call this factor “mindfulness and acceptance.” The second factor included 14 items with high (>0.45) loadings and low (<0.32) cross-loadings that were described in the theoretical model (Hayes et al., 2006) under the “commitment and behavior change” label, which we will also use to denote factor 2.

### FIT-18 questionnaire

Because the FIT-60 questionnaire included only two dimensions reflecting internally consistent individual differences that were distinguished from the other factor, we examined whether this would still be the case if we would select three items from each process included in the two dimensions. The selection process is described in the Supplemental File.

A new principal axis factoring was done with the 18 resulting items (Table 2). With this large sample size, both the Kaiser–Meyer–Olkin measure of sampling adequacy of 0.94 and Bartlett’s test of sphericity (χ^2^ = 8425, *p* < 0.001) indicated that factor analysis was appropriate. The eigenvalues of the two factors were 7.0 (39.0%) and 1.2 (6.8%) with a total explained variance of 45.8%. The Pearson correlation between the two factors was 0.57. Factor loadings of items 24, 2, and 27 were below 0.50, and the loading of item 50 was even below 0.45.

**Table 2.** Pattern matrix with factor loadings of the FIT-18 questionnaire (*N* = 2060).

	Factor	
	1	2
Items factor 1: Mindfulness and acceptance		
42R. I tend to react very strongly to my negative thoughts (Defusion)	**0.83**	−0.10
43R. I disapprove of myself when I have irrational ideas (Present)	**0.78**	−0.11
58R. I get upset with myself for having certain thoughts (Defusion)	**0.78**	−0.03
57R. It’s such a struggle to let go of upsetting thoughts even when I know that letting go would be helpful (Defusion)	**0.76**	0.02
60R. I think some of my emotions are bad or inappropriate and I shouldn’t feel them (Present)	**0.72**	0.01
38R. I believe some of my thoughts are abnormal or bad and I shouldn’t think that way (Present)	**0.69**	0.03
53R. I’m afraid of my feelings (Acceptance)	**0.65**	0.17
45R. Emotions cause problems in my life (Acceptance)	**0.64**	0.11
26R. If I allow painful feelings to arise, I am afraid they will not go away (Acceptance)	**0.61**	0.13
23R. I suffer from a negative self-image (Self)	**0.58**	0.17
24R. When I’m doing something wrong, I blame myself (Self)	**0.50**	−0.07
2R. I often feel limited by all that I feel I must do (Self)	**0.46**	0.16
Items factor 2: Commitment and behavior change		
40. I am on my way to fulfill my goals and dreams (Commitment)	−0.08	**0.72**
48. I enjoy taking on new challenges (Commitment)	−0.02	**0.70**
37. I find my life valuable (Values)	0.16	**0.66**
12. If I’m failing at something, I push through and try to tackle it in a different way (Commitment)	−0.00	**0.57**
27. There are some things I do that are important to me (Values)	0.03	**0.47**
50. I find support in the people around me (Values)	0.12	0.44

Extraction Method: Principal Axis Factoring; Rotation Method: Oblimin with Kaiser Normalization. Factor loading larger than |0.45| are indicated in bold. Processes: Acceptance, Defusion (cognitive defusion), Present (contact with the present moment), Self (self-as-context), Values, Commitment (committed action). R behind the item number indicates that item scores were reversed before they were entered in factor analysis.

**Figure 1.**
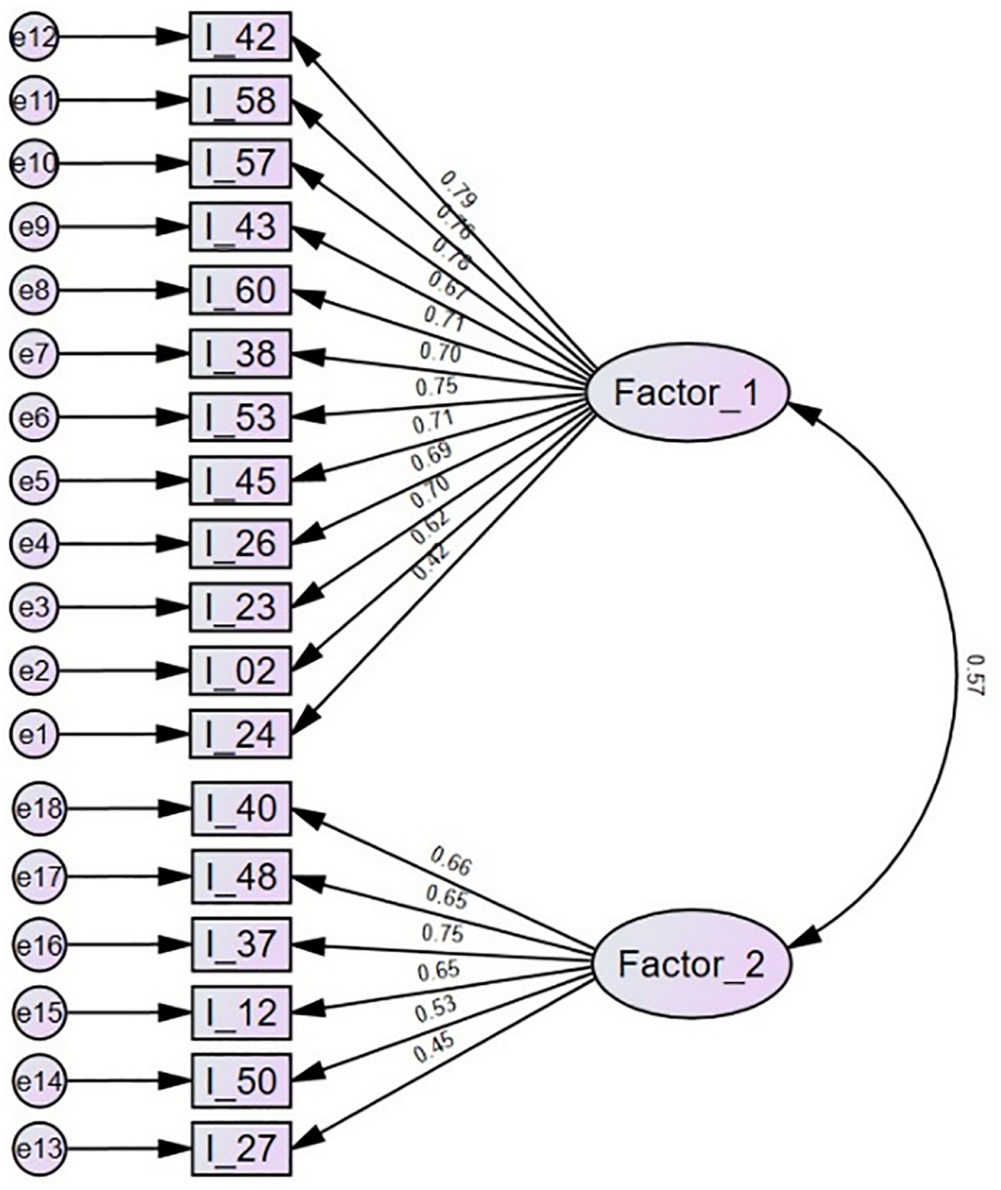
Factor loadings (standardized regression coefficients) of confirmatory factor analysis including items 42–24 for the “mindfulness and acceptance” dimension (Factor_1) and items 40–27 for the “commitment and behavior change” dimension (Factor_2).

Figure 1 shows the results of confirmatory factor analysis. For factor 1, many loadings were above or close to 0.70, but item 24 showed a poor loading. Only one item of factor 2 loaded above 0.70 with particularly low values for the items 50 and 27. The item loadings were roughly similar to those of exploratory factor analysis. The covariance between the factors (0.57) was high and about similar to the correlation of factors in exploratory factor analysis (0.59). Goodness of fit values of the confirmatory factor analysis were: χ^2^ = 789.03 (df = 134, *p* < 0.001, χ^2^/df = 5.89), RMSEA = 0.069 (confidence interval 0.64–0.74), SRMR = 0.046, CFI = 0.921, PNFI = 0.794. These values reflect that the fit of the model was borderline, that is, just good enough (SRMR, CFI, and PNFI) and just not good enough (sample-size adjusted χ^2^/df, RMSEA) to be considered adequate.

In the total sample, Cronbach’s alphas of the “mindfulness and acceptance” and “commitment and behavior change” factors were 0.92 and 0.78. The means (SD, range) of the scores on factor 1, factor 2, and the total score were 44.0 (15.5, 0–72), 26.7 (5.7, 0–36), and 70.7 (19.0, 11–108), respectively. Their score distributions were normal (skewness and kurtosis <|0.66|).

### Preservation of health in case of somatic symptoms

Linear regression analyses were done to examine whether the two dimensions of psychological flexibility might preserve mental well-being and physical functioning when having somatic symptoms. The Pearson correlations between all variables of the regression analyses are shown in Table 3. The correlations between the three health variables (mental well-being, physical functioning, and symptom severity) and the correlations between mental well-being and the two dimensions of psychological flexibility were large. Correlations of physical functioning and symptom severity with the two dimensions of psychological flexibility were medium. Correlations of demographic variables with the other variables were mostly small.

**Table 3.** Pearson correlations between mental well-being, physical functioning, and symptom severity, the “mindfulness and acceptance” and “commitment and behavior change” dimensions of psychological flexibility, and demographic variables (*N* = 2060).

Variables	1	2	3	4	5
1. Mental well-being (SF-36)					
2. Physical functioning (SF-36)	0.58***				
3. Symptom severity (SF-36)	−0.68***	−0.82***			
4. Mindfulness and acceptance (FIT-18)	0.59***	0.28***	−0.40***		
5. Commitment and behavior change (FIT-18)	0.51***	0.37***	−0.46***	0.51***	
6. Gender^a^	−0.20***	0.20***	0.26***	−0.16***	−0.07**
7. Age	0.05^*^	−0.16***	−0.02	0.17***	0.01
8. Education level^b^	0.16***	0.28***	−0.25***	0.16***	0.19***

SF-36 = Rand Short form-36, FIT-18 = Flexibility index test-18.

a Gender: 0 = men, 1 = women.

b Education level: 0 = lower general secondary education or lower; 1 = higher general secondary education or higher.

* *p* <  0.05. ** *p* <  0.01. *** *p* <  0.001.

#### Mental well-being

**Figure 2.**
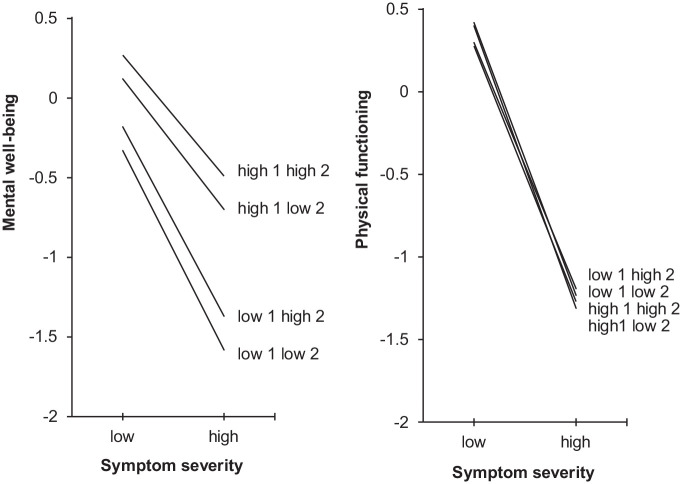
Regression lines of mental well-being and physical functioning (standard deviation from the norm) on y-axis as a function of low (−1 SD) and high (+1 SD) symptom severity (*x*-axis) for people with low (−1 SD) or high (+1 SD) “mindfulness and acceptance” (1) and low or high “commitment and behavior change” (2), while controlling for gender, age, and education level. Standard errors of measurement were 0.022 for mental well-being and 0.021 for physical functioning.

Results of the regression analyses are shown in Table 4 and Figure 2. The linear regression model showed 60.3% shared variance between the set of predictor variables and mental well-being (*F* = 386.9, *p* < 0.001). All main variables were associated with mental well-being (all *p*-values <0.001): Symptom severity (β = −0.498), “mindfulness and acceptance” (β = 0.330), and “commitment and behavioral change” (β = 0.091). Thus, while taking account of the symptom severity and the other psychological flexibility dimension, “mindfulness and acceptance” as well as “commitment and behavioral change” were additively associated with better mental health.

**Table 4.** Results of linear regression analysis examining the association of mental well-being and physical functioning (SF-36) with demographic variables, symptom severity (pain & fatigue, SF-36), the “mindfulness and acceptance” (M&A) and “commitment and behavior change” (C&BC) dimensions of psychological flexibility (FIT-18), and the two-way interaction of symptom severity with the two psychological flexibility dimensions (*N* = 2049).

	*b*	(SE)	β	*t*	*p*
Mental well-being					
Constant	−0.378	0.066		−5.76	<0.001
Gender^*^	−0.044	0.037	−0.017	−1.19	0.24
Age	−0.001	0.001	−0.019	−1.29	0.20
Education level^†^	−0.092	0.031	−0.044	−2.99	0.003
Symptom severity	−0.518	0.018	−0.498	−39.62	<0.001
M&A	0.022	0.001	0.330	19.16	<0.001
C&BC	0.016	0.003	0.091	5.02	<0.001
Symptom severity × M&A	0.007	0.001	0.113	6.73	<0.001
Symptom severity × C&BC	0.003	0.003	0.016	0.90	0.37
Physical functioning					
Constant	−0.040	0.053		−0.76	0.45
Gender^a^	0.010	0.030	0.004	0.34	0.74
Age	−0.010	0.001	−0.156	−12.60	<0.001
Education level^b^	0.108	0.025	0.054	4.32	<0.001
Symptom severity	−0.825	0.014	−0.828	−58.04	<0.001
M&A	−0.002	0.001	−0.027	−1.82	0.07
C&BC	−0.004	0.003	−0.023	−1.47	0.14
Symptom severity × M&A	−0.001	0.001	−0.019	−1.34	0.18
Symptom severity × C&BC	0.007	0.002	0.047	3.16	0.002

*b*: unstandardized regression coefficient; SE: standard error; β: standardized regression coefficient.

a Gender: 0 = men, 1 = women.

b Education level: 0 = lower general secondary education or lower; 1 = higher general secondary education or higher.

The interaction of symptom severity with “mindfulness and acceptance” was significantly associated with mental well-being (β = 0.133, *p* < 0.001). As shown by the regression lines in Figure 2, for respondents with high levels of “mindfulness and acceptance” the association between symptom severity and mental well-being was less strong than for those with low levels. The effects size difference on mental well-being for “mindfulness and acceptance” between respondents with low (−1 SD) symptom severity was small (*d* = 0.45), while it was large (*d* = 0.87) for those with high symptom severity. The effect size difference for respondents with low versus high “mindfulness and acceptance” was small (*d* = 0.42).

#### Physical functioning

The linear regression model showed 71.4% shared variance between the set of predictor variables and physical functioning (*F* = 635.3, *p* < 0.001). As shown in Table 4 and Figure 2, symptom severity accounted for virtually all variance in physical functioning. Thus, while taking account of the symptom severity and the other psychological flexibility dimension, neither “mindfulness and acceptance” nor “commitment and behavioral change” were associated with physical functioning.

The interaction of symptom severity with “commitment and behavior change” was significantly associated with physical functioning (β = 0.047, *p* = 0.002). The effect size difference on physical functioning for “commitment and behavior change” between respondents with low (*d* = 0.12) and high (*d* = 0.04) symptom severity was trivial. Thus, also the effect size difference for respondents with low versus high “commitment and behavior change” was trivial (*d* = 0.08).

## Discussion

This study yielded the FIT-18 questionnaire, a brief 18-item version of the FIT-60 questionnaire. Exploratory factor-analysis generated two factors with a clear allocation of items, belonging to the two predefined overarching dimensions of psychological flexibility: “mindfulness and acceptance” and “commitment and behavior change,” respectively. The internal consistency of the two dimensions (Cronbach’s alpha of 0.92 and 0.78) was good. The structural validity was borderline (neither weak nor strong). While taking account of all variables in the model, symptom severity, “mindfulness and acceptance” and “commitment and behavior change” were additively associated with mental well-being. Furthermore, moderator analysis showed that the association between symptom severity and mental well-being was higher in people with low than high “mindfulness and acceptance.” The dimensions of psychological flexibility were not associated with physical functioning.

The six intended dimensions of the FIT-60 could not be derived in factor analysis. Perhaps the interdependence of the processes prevented us from finding all six processes. Ideally, an exhaustive database of items should be used to derive items that are correlated with other items of the same dimension, but not with items of other dimensions. After the start of data collection in our study, such a method was adopted when developing the 60-item Multidimensional Psychological Flexibility Inventory (MPFI, Rolffs et al., 2018). That study even derived 12 independent dimensions, that represented both the positive and the negative version of each of the six processes comprising psychological flexibility; for instance, “acceptance” as well as the opposite construct “experiential avoidance,” or “committed action” as well as “inaction.” Of note, the pattern matrix with factor loadings of the MPFI clearly showed that these six negative versions are not simply the opposite poles of the six positive psychological flexibility versions. Moreover, it was shown that variables could vary independently, that is, patients could show improvement on specific dimensions of flexibility without showing improvement on all dimensions of flexibility, and without showing similar improvements on the corresponding dimensions of inflexibility (Rogge et al., 2019; Rolffs et al., 2018). Thus, the validity tests supported a 12-factor solution instead of a 6-factor solution. Therefore, the MPFI appears a better instrument than the FIT-60 for a researcher or clinician who wants to use self-report questionnaires to assess all processes of both psychological flexibility and psychological *in*flexibility.

The FIT-18 includes items of the two overarching dimensions of psychological flexibility. The first factor included only negatively worded items representing the four processes of “mindfulness and acceptance” and the second factor included only positively worded items of the two processes of “commitment and behavior change.” Thus, the first factor includes only psychological *in*flexibility items and the second factor only psychological *flexibility* items. The results of exploratory factor analysis and confirmatory factor analysis were largely similar. Nevertheless, the result of confirmatory factor analysis was a little weaker in terms of acceptance of the model. This is common, because the confirmatory model is more restrictive (Lorenzo-Seva and Ferrando, 2020). Moreover, we did not want to include very similar items within the factors and took care that three items of every assumed process of psychological flexibility were included. This may have increased the content validity because all six assumed underlying processes were evenly represented in the final solution, but it will have reduced the structural validity. While the grouping of items in factor analysis clearly discriminated between the two predefined overarching factors of psychological flexibility, there are challenges for interpretation. Three somewhat overlapping challenges are discussed: response bias, approach-avoidance, and affect.

First, response bias may hamper interpretation. Besides in terms of content, the two dimensions showed a semantic difference. The first dimension includes exclusively negatively worded items and the second positively worded items. This wording may cue the respondent to give specific answers such as agreeing with positive worded items and disagreeing with negatively worded items (acquiescence), which could be the cause of getting two factors (e.g. Lindwall et al., 2012). The development of the MPFI showed that negatively and positively worded items of the same processes were included in distinct factors (Rolffs et al., 2018). This can suggest that the content of the construct changes when the wording changes as well as that the changed wording triggers response bias. Ideally, it should be tried to include an equal amount of positively and negatively worded items in each dimension such as has been done with the Big Five Inventory-2 (Soto and John, 2017). This could not be done with the FIT-60, because too few positively worded items are included in this questionnaire.

Second, 2-factor solutions of questionnaires often differentiate between what Pribram (1981) called “out of motion” (e-motion) and motivation (in-motion) processes and what has been described as avoidance and approach temperaments (Elliot and Thrash, 2002) or behavioral inhibition versus behavioral activation systems (Carver and White, 1994). These emotional “stop” and motivational “go” processes have been linked to differential neurological systems. To a certain extent, experiential avoidance is reflected in the items of the “mindfulness and acceptance” dimension and motivational approach behavior in the items of the “commitment and behavior change” dimension.

Third, and related to the other two interpretations, two-factor solutions of questionnaires often differentiate negative and positive affectivity (Tellegen et al., 1999) or the personality factors neuroticism and extraversion. The first questionnaire to measure psychological flexibility was the Acceptance and Action Questionnaire (AAQ-II; Bond et al., 2011). This questionnaire assessed a single dimension called experiential avoidance or psychological inflexibility. Critiques indicated at the overlap between this questionnaire and psychological distress (Tyndall et al., 2019; Wolgast, 2014). The ambition of questionnaires of psychological (in)flexibility is to assess processes and skills that may influence mood, but it is an inherent problem of self-report measures that they contain a substantial pervasive mood disposition of negative affectivity (Watson and Pennebaker, 1989). Therefore, to get insight in what FIT-18 actually measures, future research of construct validity should assess associations of the two FIT-18 dimensions with self-reports of approach-avoidance (e.g. the BIS/BAS scales, Carver and White, 1994) and positive and negative affectivity (e.g. The Positive and Negative Affect Schedule (Watson et al., 1988)) to test divergent validity. Moreover, it should be examined whether the FIT-18 dimensions are more sensitive to change in response to interventions that target psychological flexibility, than these other self-report measures.

In examining the potential applicability of the FIT-18, we observed that physical functioning was strongly associated with symptom severity, but not with the psychological flexibility dimensions. The interaction between symptom severity and “commitment & behavior change” was significantly associated with physical functioning, but the effect size was trivial. In contrast, the association with mental well-being indicated a role for psychological flexibility. While adjusting for symptom severity, both psychological flexibility dimensions were additively associated with mental well-being. Although such associations have sometimes been presented as showing that psychological flexibility preserves mental well-being (Dawson and Golijani-Moghaddam, 2020; McCracken et al., 2021), this inference from a cross-sectional observation is too strong. Other mechanisms may explain the observations; for instance, that mental well-being influences psychological flexibility, a third variable influences both mental well-being and psychological flexibility, or that the associations reflect mutual influences, overlap between items, or confounding by answer tendencies. Our interaction of symptom severity with the “mindfulness and acceptance” dimension gave a stronger indication. In agreement with previous observations of moderation (Gloster et al., 2017; Leonidou et al., 2019; Pleman et al., 2019), the significant interaction suggested that psychological flexibility had a stronger role in preserving mental well-being in case of high than low symptom severity. Overall, the patterns of associations with health variables indicate that the two dimensions of psychological flexibility have a different meaning. Longitudinal and clinical experimental research is needed to get a more thorough understanding of the directionality of associations and the changeability of variables.

A strength of the current study is the large sample including many people with severe pain and fatigue for whom psychological flexibility might be relevant. Furthermore, whereas in other studies measurement of psychological flexibility was often limited to one or a few processes underlying psychological flexibility, this study used items encompassing all six processes. In this stage of questionnaire development, the collected data were cross-sectional and the first analysis of validity was restricted to one aspect of construct validity. An indication of applicability of the FIT-18 questionnaire was not yet obtained using repeated measures within persons. Future validation studies could also employ other self-report measures to examine convergent and divergent validity and other than self-report questionnaires, which might better reflect dynamic processes that are intended by the psychological flexibility construct. The differences between demographic variables of completers and dropouts were significant but small. The results of our study do not generalize beyond the report of self-perceived health, the participating Dutch sample, and the employed cross-sectional design.

An asset of the FIT-18 questionnaire as compared to other questionnaires is that it includes only 18 items which will have positive effects on the burden for respondents, the response rate, and the possibility to take repeated assessments across time. After and as part of more validation research, the FIT-18 might be used to (further) examine associations with existing questionnaires, measurement invariance, moderation and mediation, and sensitivity to change. It should also be examined whether the questionnaire can inform treatment by using it to screen whether a mindfulness and acceptance-based or value-based therapeutic approach is indicated for a person or both, and to monitor and evaluate the outcome of therapy. If further research confirms its validity, the FIT-18 is a succinct questionnaire that can be easily and quickly applied in research and clinical practice to get insight into the “mindfulness and acceptance” dimension of psychological inflexibility and the “commitment and behavior change” dimension of psychological flexibility.

## Supplemental Material

sj-docx-1-hpq-10.1177_13591053241239129 – Supplemental material for Dimensions of psychological flexibility and their significance in people with somatic symptoms: The 18-item Flexibility Index Test (FIT-18) Supplemental material, sj-docx-1-hpq-10.1177_13591053241239129 for Dimensions of psychological flexibility and their significance in people with somatic symptoms: The 18-item Flexibility Index Test (FIT-18) by Tim Y Koppert, Renée van Hoek and Rinie Geenen in Journal of Health Psychology
