# Supplementary material for: Dimensions of psychological flexibility and their significance in people with somatic symptoms: The 18-item Flexibility Index Test (FIT-18)
Source: J Health Psychol. 2024 Apr 2;30(7):1444–59. doi: 10.1177/13591053241239129 (PMC12166160; doi:10.1177/13591053241239129)
Supplement: sj-docx-1-hpq-10.1177_13591053241239129 – Supplemental material for Dimensions of psychological flexibility and their significance in people with somatic symptoms: The 18-item Flexibility Index Test (FIT-18) [file sj-docx-1-hpq-10.1177_13591053241239129.docx]

**Supplementary file**

Of the article: Dimensions of psychological flexibility and their significance in people with somatic symptoms: The 18-item Flexibility Index test (FIT-18)

This supplementary file includes supplementary figure F1, supplementary table T2, supplementary table T3 with accompanying text and both an English and Dutch version of the FIT-18 questionnaire.

**Figure S1.** Flowchart of the data that were collected in 2018 and 2020


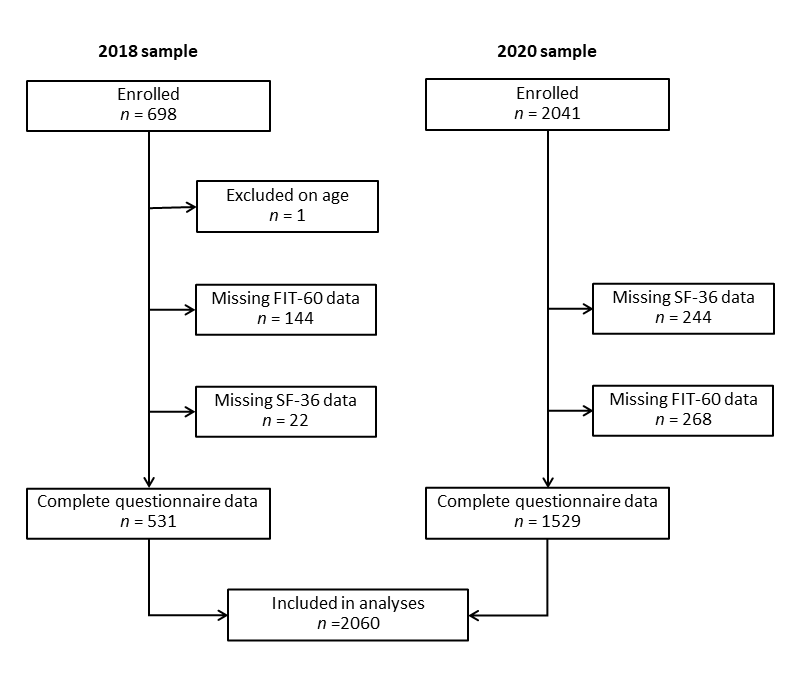


Note: FIT-60=Flexibility Index Test-60 (Batink et al., 2012), SF-36=RAND Short Form-36 (Vanderzee et al., 1996). The questionnaire was administered in the same way, but in a different order in 2018 (first FIT-60) and 2020 (first SF-36).

**Table S1**. Pattern matrix with factor loadings of a six-factor solution

|  | **Factor** | | | | | |
| --- | --- | --- | --- | --- | --- | --- |
|  | 1 | 2 | 3 | 4 | 5 | 6 |
| 60R. I think some of my emotions are bad or inappropriate and I shouldn’t feel them  (Present) | **.74** | .01 | .07 | .08 | -.06 | -.01 |
| 38R. I believe some of my thoughts are abnormal or bad and I shouldn’t think that way (Present) | **.71** | .04 | .03 | .05 | -.03 | -.02 |
| 52R. I am sometimes frightened by the thoughts I have (Defusion) | **.66** | .07 | -.06 | .11 | .04 | -.03 |
| 53R. I'm afraid of my feelings (Acceptance) | **.63** | .12 | .08 | .11 | .03 | -.03 |
| 43R. I disapprove of myself when I have irrational ideas (Present) | **.60** | -.01 | .01 | .04 | .18 | .01 |
| 58R. I get upset with myself for having certain thoughts (Defusion) | **.51** | .05 | -.11 | .09 | .26 | -.09 |
| 35R. I worry about not being able to control my worries and feelings (Acceptance) | **.50** | -.09 | .12 | .05 | .11 | -.02 |
| 45R. Emotions (for instance, anger, sadness) cause problems in my life (Acceptance) | **.50** | .09 | -.03 | .18 | .13 | -.05 |
| 26R. If I allow painful feelings to arise, I am afraid they will not go away (Acceptance) | **.48** | .08 | .12 | .07 | .16 | -.08 |
| 42R. I tend to react very strongly to my negative thoughts (Defusion) | **.47** | .00 | -.07 | .09 | .35 | -.09 |
| 57R. It’s such a struggle to let go of upsetting thoughts even when I know that letting go would be helpful (Defusion) | **.46** | .09 | -.06 | .07 | .33 | -.10 |
| 10. It's OK if I remember something unpleasant (Acceptance) | .36 | .10 | .18 | .12 | .02 | .04 |
| 31R. My painful experiences and memories make it difficult for me to live a life that I would value (Acceptance) | .36 | .17 | -.05 | .34 | .08 | -.11 |
| 9R. My thoughts cause me distress or emotional pain (Defusion) | .34 | .10 | -.13 | .19 | .20 | -.24 |
| 29R. I tend to make pain worse in my mind (Defusion) | .33 | .05 | -.02 | .02 | .22 | -.18 |
| 22R. I will do my best not to have to experience negative things (Acceptance) | .32 | -.02 | .03 | -.13 | .01 | -.07 |
| 59. I aim to do new things (Commitment) | -.08 | **.70** | .08 | .00 | .12 | .02 |
| 40. I am on my way to fulfill my goals and dreams (Commitment) | -.05 | **.67** | .00 | .13 | .11 | -.03 |
| 48. I enjoy taking on new challenges (Commitment) | .01 | **.63** | .12 | .06 | .12 | -.01 |
| 34. My work and/or study plays a significant role in my life (Values) | .09 | **.59** | -.12 | -.02 | -.12 | .11 |
| 11. I regularly make concrete plans for the future (Commitment) | -.01 | **.54** | .09 | .06 | -.04 | -.08 |
| 13. I like to go to my work (Commitment) | .10 | **.54** | -.12 | .10 | -.01 | .00 |
| 12. If I’m failing at something, I push through and try to tackle it in a different way (Commitment) | .01 | **.47** | .20 | .01 | -.09 | -.26 |
| 4. If I want to do something, I go for it (Commitment) | .00 | **.45** | .18 | .00 | -.11 | -.25 |
| 47. I do several things that are important to me (Commitment) | .03 | .40 | .12 | .36 | .04 | -.05 |
| 5. I am capable of dividing my long-term goals into short term possibilities (Commitment) | -.02 | .35 | .23 | .02 | .00 | -.35 |
| 36. I’m good at finding words to describe my feelings (Present) | .03 | .04 | **.69** | -.03 | .10 | -.14 |
| 49. I can well describe what I experience with my senses, such as what I hear, see and smell (Present) | .02 | .06 | **.55** | .05 | .02 | -.13 |
| 44. I can easily put my beliefs, opinions, and expectations into words (Present) | .02 | .17 | **.53** | -.01 | .15 | -.15 |
| 18R. It’s hard for me to find the words to describe what I’m thinking (Present) | .14 | .04 | **.**35 | .03 | .12 | -.35 |
| 14. I am willing to fully allow my fears (Acceptance) | .14 | .07 | .34 | .01 | .06 | .09 |
| 30. I find it easy to look at my thoughts from a different perspective (Defusion) | .14 | .23 | .27 | .01 | .22 | .01 |
| 20. I watch my feelings without getting lost in them (Present) | .19 | .16 | .26 | .08 | .22 | .00 |
| 51. The thoughts I have about myself do not define who I am (Self) | .03 | -.05 | .26 | .07 | -.06 | .11 |
| 19. I realize that my self-image doesn't say much about me as a person (Self) | -.08 | -.11 | .17 | .02 | -.10 | .15 |
| 50. I find support in the people around me  (Values) | .04 | .03 | .07 | **.66** | .03 | .07 |
| 8. I have enough friends (Values) | .05 | .12 | -.02 | **.55** | .06 | .10 |
| 37. I find my life valuable (Values) | .04 | .22 | .13 | **.51** | .17 | -.03 |
| 55. I find my friends and/or family important (Values) | .03 | .09 | .04 | **.46** | -.08 | .12 |
| 6. My life is well balanced (Values) | -.07 | .19 | -.03 | **.46** | .20 | -.25 |
| 21. When I am at home, I feel comfortable (Values) | .13 | -.04 | .13 | .40 | -.09 | -.16 |
| 1R. Worries get in the way of my success (Acceptance) | .08 | -.04 | -.05 | .39 | .13 | -.39 |
| 41. I regularly spend time on my hobbies (Values) | -.14 | .13 | .04 | .38 | .14 | -.14 |
| 46R. I am detached from my environment (Self) | .23 | -.04 | -.03 | .33 | -.14 | -.13 |
| 54. My thoughts and feelings don't get in the way of how I want to live my life (Acceptance) | .20 | .03 | .12 | .27 | .08 | -.05 |
| 27. There are some things I do that are important to me (Values) | .08 | .23 | .17 | .24 | -.07 | -.04 |
| 25. I realize that the things I do, I have chosen myself (Values) | .02 | .16 | .15 | .20 | .02 | -.05 |
| 32R. If someone makes a nasty comment, it can bother me for a long time (Defusion) | .16 | .12 | -.03 | -.05 | **.69** | .05 |
| 39R. Some words can hurt me deeply (Defusion) | .15 | .06 | -.08 | -.06 | **.58** | .04 |
| 24R. When I’m doing something wrong, I blame myself (Self) | .08 | -.04 | .04 | -.03 | **.58** | .00 |
| 33. I do not always have to do the things right for myself (Self) | -.02 | -.09 | .18 | .16 | **.49** | .08 |
| 23R. I suffer from a negative self-image (Self) | .14 | .09 | .00 | .20 | **.48** | -.12 |
| 17R. I think I should always be nice (Self) | .08 | -.09 | .06 | -.06 | **.46** | -.12 |
| 3. I can have negative thoughts about myself and at the same time know that I am okay (Self) | -.15 | -.01 | .20 | -.06 | -.26 | .21 |
| 56R. When I compare myself to other people, it seems that most of them are in better control of their lives than I am (Self) | .21 | .06 | .03 | .26 | .26 | -.25 |
| 15R. I find it difficult to stay focused on what’s happening in the present (Present) | .19 | -.05 | .05 | -.03 | -.01 | **-.61** |
| 16R. I am easily distracted (Present) | .19 | .01 | .03 | -.09 | .00 | **-.52** |
| 7R. I find it hard to keep on focusing on goals (Commitment) | .07 | .16 | .00 | -.00 | -.05 | **-.51** |
| 28R. I tend to get very entangled in my thoughts (Defusion) | .19 | .00 | .01 | .21 | .19 | -.43 |
| 2R. I often feel limited by all that I feel I must do (Self) | -.01 | -.04 | -.09 | .32 | .32 | -.40 |

Notes. Extraction Method: Principal Axis Factoring, Rotation Method: Oblimin with Kaiser Normalization.

Factor loading larger than |.45| are indicated in bold.

Psychological flexibility processes: Acceptance, Defusion (cognitive defusion), Present (contact with the present moment), Self (self-as-context), Values, Commitment (committed action).

R behind the item number indicates that item scores were reversed before entered in factor analysis

**Selection of 18 items from 60 items**

In selecting items, we first looked at the factor loadings of factor analysis forcing a 2-factor solution (high on the primary factor and low on the other factor). Items that overlapped too much in contents with other items were not chosen.

As a reflection of ‘acceptance’, the three highest loading items on the first factor were chosen: 53R, 45R, 26R.

For ‘cognitive defusion’, the three highest loading items on the first factor were chosen: 42R, 57R, 58R.

For ‘contact with the present moment’, the three highest loading items on the first factor were chosen: 43R, 38R, 60R.

For ‘self-as-context’, three of the four highest loading items on the first factor were chosen: 23R, 24R, 2R; item 56R was not chosen because its formulation was considered complex.

For ‘values’, the three highest loading items on the second factor were chosen: 37, 27, 50.

For ‘committed action’, three of the four highest loading items on the second factor were chosen: 48, 40, 12; item 47 was not chosen, because its formulation overlapped too much with item 27.

| **Table S2**. Pattern matrix with factor loadings of a two-factor solution. Three asterisks (***) mark items that were selected for the FIT-18 questionnaire | | | | |
| --- | --- | --- | --- | --- |
|  | | Factor | | |
|  |  | 1 | | 2 |
| 42R. I tend to react very strongly to my negative thoughts (Defusion) *** | **.79** | | -.03 | |
| 57R. It’s such a struggle to let go of upsetting thoughts even when I know that letting go would be helpful (Defusion) *** | **.76** | | .05 | |
| 58R. I get upset with myself for having certain thoughts (Defusion) *** | **.75** | | .02 | |
| 43R. I disapprove of myself when I have irrational ideas (Present) *** | **.70** | | -.02 | |
| 32R. If someone makes a nasty comment, it can bother me for a long time (Defusion) | **.66** | | -.04 | |
| 52R. I am sometimes frightened by the thoughts I have (Defusion) | **.65** | | .10 | |
| 9R. My thoughts cause me distress or emotional pain (Defusion) | **.64** | | .15 | |
| 23R. I suffer from a negative self-image (Self) *** | **.63** | | .16 | |
| 38R. I believe some of my thoughts are abnormal or bad and I shouldn’t think that way (Present) *** | **.63** | | .10 | |
| 60R. I think some of my emotions are bad or inappropriate and I shouldn’t feel them (Present) *** | **.62** | | .12 | |
| 53R. I'm afraid of my feelings (Acceptance) *** | **.61** | | .23 | |
| 45R. Emotions (for instance, anger, sadness) cause problems in my life (Acceptance) *** | **.61** | | .17 | |
| 26R. If I allow painful feelings to arise, I am afraid they will not go away (Acceptance) *** | **.61** | | .17 | |
| 28R. I tend to get very entangled in my thoughts (Defusion) | **.61** | | .19 | |
| 39R. Some words can hurt me deeply (Defusion) | **.59** | | -.12 | |
| 29R. I tend to make pain worse in my mind (Defusion) | **.58** | | .04 | |
| 56R. When I compare myself to other people, it seems that most of them are in better control of their lives than I am (Self) | **.58** | | .25 | |
| 35R. I worry about not being able to control my worries and feelings (Acceptance) | **.55** | | -.06 | |
| 24R. When I’m doing something wrong, I blame myself (Self) *** | **.55** | | -.12 | |
| 2R. I often feel limited by all that I feel I must do (Self) *** | **.54** | | .15 | |
| 17R. I think I should always be nice (Self) | **.52** | | -.14 | |
| 15R. I find it difficult to stay focused on what’s happening in the present (Present) | **.50** | | .11 | |
| 3. I can have negative thoughts about myself and at the same time know that I am okay (Self) # | -.49 | | .07 | |
| 31R. My painful experiences and memories make it difficult for me to live a life that I would value (Acceptance) | **.49** | | .35 | |
| 1R. Worries get in the way of my success (Acceptance) | **.47** | | .24 | |
| 16R. I am easily distracted (Present) | .44 | | .06 | |
| 18R. It’s hard for me to find the words to describe what I’m thinking (Present) | .40 | | .29 | |
| 33. I do not always have to do the things right for myself (Self) | .36 | | .04 | |
| 20. I watch my feelings without getting lost in them (Present) | .33 | | .31 | |
| 22R. I will do my best not to have to experience negative things (Acceptance) | .32 | | -.08 | |
| 10. It's OK if I remember something unpleasant (Acceptance) | .32 | | .25 | |
| 54. My thoughts and feelings don't get in the way of how I want to live my life (Acceptance) | .31 | | .27 | |
| 7R. I find it hard to keep on focusing on goals (Commitment) | .28 | | .24 | |
| 19. I realize that my self-image doesn't say much about me as a person (Self) | -.24 | | .01 | |
| 46R. I am detached from my environment (Self) | .22 | | .21 | |
| 47. I do several things that are important to me (Commitment) | .09 | | **.68** | |
| 48. I enjoy taking on new challenges (Commitment) *** | .04 | | **.67** | |
| 40. I am on my way to fulfill my goals and dreams (Commitment) *** | -.01 | | **.67** | |
| 59. I aim to do new things (Commitment) | -.05 | | **.65** | |
| 48. I enjoy taking on new challenges (Commitment) *** | .05 | | **.66** | |
| 12. If I’m failing at something, I push through and try to tackle it in a different way (Commitment) *** | .03 | | **.61** | |
| 11. I regularly make concrete plans for the future (Commitment) | -.05 | | **.60** | |
| 37. I find my life valuable (Values) *** | .25 | | **.59** | |
| 4. If I want to do something, I go for it (Commitment) | -.01 | | **.57** | |
| 5. I am capable of dividing my long-term goals into short term possibilities (Commitment) | .13 | | **.52** | |
| 27. There are some things I do that are important to me (Values) *** | .04 | | **.49** | |
| 13. I like to go to my work (Commitment) | .05 | | **.49** | |
| 50. I find support in the people around me (Values) *** | .13 | | **.47** | |
| 6. My life is well balanced (Values) | .31 | | **.47** | |
| 34. My work and/or study plays a significant role in my life (Values) | -.13 | | .44 | |
| 44. I can easily put my beliefs, opinions, and expectations into words (Present) | .19 | | .44 | |
| 8. I have enough friends (Values) | .12 | | .43 | |
| 49. I can well describe what I experience with my senses, such as what I hear, see and smell (Present) | .09 | | .41 | |
| 36. I’m good at finding words to describe my feelings (Present) | .15 | | .40 | |
| 55. I find my friends and/or family important (Values) | -.04 | | .40 | |
| 41. I regularly spend time on my hobbies (Values) | .11 | | .39 | |
| 25. I realize that the things I do, I have chosen myself (Values) | .08 | | .37 | |
| 21. When I am at home, I feel comfortable (Values) | .19 | | .34 | |
| 30. I find it easy to look at my thoughts from a different perspective (Defusion) | .27 | | .33 | |
| 14. I am willing to fully allow my fears (Acceptance) | .10 | | .23 | |
| 51. The thoughts I have about myself do not define who I am (Self) | -.09 | | .14 | |

Notes. Extraction Method: Principal Axis Factoring, Rotation Method: Oblimin with Kaiser Normalization.

Factor loading larger than |.45| are indicated in bold.

Psychological flexibility processes: Acceptance, Defusion (cognitive defusion), Present (contact with the present moment), Self (self-as-context), Values, Commitment (committed action).

R behind the item number indicates that item scores were reversed before entered in factor analysis

# Item 3 was not included in the factor because the loading was negative.

**FIT-18 (English version)**

This questionnaire consists of 18 statements. Please read each statement carefully and indicate to what extent the following statements apply to you. You can choose any number between 0 (*strongly disagree*) and 6 (*strongly agree*). The higher the number, the more the statement applies to you. Make sure to answer all statements.

To indicate your answer, please circle the corresponding number.

*Example*:

**Question|** I find support in the people around me.  0  1  2  3  4  5  6    

If you have circled an answer but want to alter your choice, you can correct it by drawing a line through the given answer. Then circle the desired answer

*Example*:

**Question|** I find support in the people around me.  0  1  2  3  4  5  6

Good luck completing this questionnaire!

Name: ____________________________________  Gender: M / V / X  Age: _______

Marital status: _____________ Education: ______________________ Date: _________

|  | To what extent do the following statements apply to you?  Circle the most appropriate answer.  Please do not skip statements. | **Strongly disagree** | |  | |  | |  | |  | | |  | | **Strongly agree** | |
| --- | --- | --- | --- | --- | --- | --- | --- | --- | --- | --- | --- | --- | --- | --- | --- | --- |
| 1**\|** | I believe some of my thoughts are abnormal or bad and I shouldn’t think that way | | 0 | | 1 | | 2 | | 3 | | 4 | 5 | | 6 | |  |
| 2**\|** | I find support in the people around me | | 0 | | 1 | | 2 | | 3 | | 4 | 5 | | 6 | |  |
| 3**\|** | There are some things I do that are important to me | | 0 | | 1 | | 2 | | 3 | | 4 | 5 | | 6 | |  |
| 4**\|** | Emotions (for instance, anger, sadness) cause problems in my life | | 0 | | 1 | | 2 | | 3 | | 4 | 5 | | 6 | |  |
| 5**\|** | I find my life valuable | | 0 | | 1 | | 2 | | 3 | | 4 | 5 | | 6 | |  |
| 6**\|** | I am on my way to fulfill my goals and dreams | | 0 | | 1 | | 2 | | 3 | | 4 | 5 | | 6 | |  |
| 7**\|** | I think some of my emotions are bad or inappropriate and I shouldn’t feel them | | 0 | | 1 | | 2 | | 3 | | 4 | 5 | | 6 | |  |
| 8**\|** | When I’m doing something wrong, I blame myself | | 0 | | 1 | | 2 | | 3 | | 4 | 5 | | 6 | |  |
| 9**\|** | If I allow painful feelings to arise, I am afraid they will not go away | | 0 | | 1 | | 2 | | 3 | | 4 | 5 | | 6 | |  |
| 10**\|** | I'm afraid of my feelings | | 0 | | 1 | | 2 | | 3 | | 4 | 5 | | 6 | |  |
| 11**\|** | I enjoy taking on new challenges | | 0 | | 1 | | 2 | | 3 | | 4 | 5 | | 6 | |  |
| 12**\|** | If I’m failing at something, I push through and try to tackle it in a different way | | 0 | | 1 | | 2 | | 3 | | 4 | 5 | | 6 | |  |
| 13**\|** | I disapprove of myself when I have irrational ideas | | 0 | | 1 | | 2 | | 3 | | 4 | 5 | | 6 | |  |
| 14**\|** | I suffer from a negative self-image | | 0 | | 1 | | 2 | | 3 | | 4 | 5 | | 6 | |  |
| 15**\|** | It’s such a struggle to let go of upsetting thoughts even when I know that letting go would be helpful | | 0 | | 1 | | 2 | | 3 | | 4 | 5 | | 6 | |  |
| 16**\|** | I get upset with myself for having certain thoughts | | 0 | | 1 | | 2 | | 3 | | 4 | 5 | | 6 | |  |
| 17**\|** | I often feel limited by all that I feel I must do | | 0 | | 1 | | 2 | | 3 | | 4 | 5 | | 6 | |  |
| 18**\|** | I tend to react very strongly to my negative thoughts | | 0 | | 1 | | 2 | | 3 | | 4 | 5 | | 6 | |  |

**FIT-18 (Nederlandse versie)**

Deze vragenlijst bestaat uit 18 stellingen. Lees elke stelling aandachtig door en geef daarna aan in welke mate onderstaande stellingen op u van toepassing zijn. U kunt alle cijfers tussen 0 (*helemaal oneens*) en 6 (*helemaal eens*) kiezen. Hoe hoger het cijfers, hoe meer de stelling op u van toepassing is. Let er op dat u bij alle stellingen een antwoord geeft.

Om uw antwoordkeuze aan te geven, kunt u het corresponderende cijfer omcirkelen.

*Voorbeeld*:

**Vraag|** Ik vind steun bij de mensen in mijn omgeving 0  1  2  3  4  5  6    

Indien u een verkeerd antwoord heeft omcirkeld, kunt u dit corrigeren door een streep te zetten door het gegeven antwoord. Vervolgens omcirkeld u alsnog het correcte antwoord.

*Voorbeeld*:

**Vraag|** Ik vind steun bij de mensen in mijn omgeving.   0  1  2  3  4  5  6

Succes met het invullen van deze lijst!

Naam: ____________________________________ Geslacht: M / V / X  Leeftijd:_____

Burg. staat: _________________ Opleiding: __________________ Datum: _________

|  | In welke mate zijn onderstaande stellingen van toepassing op u? Omcirkel het meest passende antwoord. Sla alstublieft geen stellingen over. | **Helemaal oneens** | |  | |  | |  | |  | | |  | | **Helemaal eens** | |
| --- | --- | --- | --- | --- | --- | --- | --- | --- | --- | --- | --- | --- | --- | --- | --- | --- |
| 1**\|** | Ik geloof dat sommige van mijn gedachten abnormaal of slecht zijn en dat ik niet zo zou moeten denken | | 0 | | 1 | | 2 | | 3 | | 4 | 5 | | 6 | |  |
| 2**\|** | Ik vind steun bij de mensen in mijn omgeving | | 0 | | 1 | | 2 | | 3 | | 4 | 5 | | 6 | |  |
| 3**\|** | Er zijn een aantal dingen die ik doe, die ik belangrijk vind | | 0 | | 1 | | 2 | | 3 | | 4 | 5 | | 6 | |  |
| 4**\|** | Emoties (zoals boosheid, verdriet) veroorzaken problemen in mijn leven | | 0 | | 1 | | 2 | | 3 | | 4 | 5 | | 6 | |  |
| 5**\|** | Ik vind mijn leven waardevol | | 0 | | 1 | | 2 | | 3 | | 4 | 5 | | 6 | |  |
| 6**\|** | Ik ben onderweg om mijn doelen en dromen te bereiken | | 0 | | 1 | | 2 | | 3 | | 4 | 5 | | 6 | |  |
| 7**\|** | Ik denk dat mijn emoties soms slecht of ongepast zijn en dat ik ze niet zou moeten voelen | | 0 | | 1 | | 2 | | 3 | | 4 | 5 | | 6 | |  |
| 8**\|** | Als ik iets niet goed doe, dan reken ik dat mezelf aan | | 0 | | 1 | | 2 | | 3 | | 4 | 5 | | 6 | |  |
| 9**\|** | Als ik pijnlijke gevoelens toelaat, dan ben ik bang dat ze niet meer verdwijnen | | 0 | | 1 | | 2 | | 3 | | 4 | 5 | | 6 | |  |
| 10**\|** | Ik ben bang voor mijn gevoelens | | 0 | | 1 | | 2 | | 3 | | 4 | 5 | | 6 | |  |
| 11**\|** | Ik vind het leuk om nieuwe uitdagingen aan te gaan | | 0 | | 1 | | 2 | | 3 | | 4 | 5 | | 6 | |  |
| 12**\|** | Als iets me niet lukt dan zet ik door, en probeer ik het op een andere manier aan te pakken | | 0 | | 1 | | 2 | | 3 | | 4 | 5 | | 6 | |  |
| 13**\|** | Ik keur mezelf af als ik rare gedachten heb | | 0 | | 1 | | 2 | | 3 | | 4 | 5 | | 6 | |  |
| 14**\|** | Ik heb last van een negatief zelfbeeld | | 0 | | 1 | | 2 | | 3 | | 4 | 5 | | 6 | |  |
| 15**\|** | Het is erg moeilijk om verontrustende gedachten los te laten, zelfs wanneer ik weet dat los laten mij zou helpen | | 0 | | 1 | | 2 | | 3 | | 4 | 5 | | 6 | |  |
| 16**\|** | Van sommige gedachten raak ik van streek | | 0 | | 1 | | 2 | | 3 | | 4 | 5 | | 6 | |  |
| 17**\|** | Ik voel me vaak beperkt door alles wat ik van mezelf moet | | 0 | | 1 | | 2 | | 3 | | 4 | 5 | | 6 | |  |
| 18**\|** | Ik heb de neiging erg sterk te reageren op mijn eigen negatieve gedachten | | 0 | | 1 | | 2 | | 3 | | 4 | 5 | | 6 | |  |
